# A Germline Variant on Chromosome 4q31.1 Associates with Susceptibility to Developing Colon Cancer Metastasis

**DOI:** 10.1371/journal.pone.0146435

**Published:** 2016-01-11

**Authors:** Sanford D. Markowitz, Nora L. Nock, Stephanie L. Schmit, Zsofia K. Stadler, Vijai Joseph, Lu Zhang, Joseph E. Willis, Peter Scacheri, Martina Veigl, Mark D. Adams, Leon Raskin, John F. Sullivan, Kelly Stratton, Jinru Shia, Nathan Ellis, Hedy S. Rennert, Christopher Manschreck, Li Li, Kenneth Offit, Robert C. Elston, Gadi Rennert, Stephen B. Gruber

**Affiliations:** 1 Department of Medicine, Case Western Reserve University, Cleveland, Ohio, United States of America; 2 Department of Epidemiology and Biostatistics, Case Western Reserve University, Cleveland, Ohio, United States of America; 3 Department of Genetics, Case Western Reserve University, Cleveland, Ohio, United States of America; 4 Department of Pathology, Case Western Reserve University, Cleveland, Ohio, United States of America; 5 Case Comprehensive Cancer Center, Case Western Reserve University, Cleveland, Ohio, United States of America; 6 Department of Preventive Medicine, Keck School of Medicine, University of Southern California, Los Angeles, California, United States of America; 7 USC Norris Comprehensive Cancer Center, Keck School of Medicine, University of Southern California, Los Angeles, California, United States of America; 8 Department of Medicine, Memorial Sloan-Kettering Cancer Center, New York, New York, United States of America; 9 Department of Pathology, Clinical Genetics Service, Memorial Sloan-Kettering Cancer Center, New York, New York, 10065, United States of America; 10 Vanderbilt Epidemiology Center, Vanderbilt University, Nashville, Tennessee, United States of America; 11 The University of Arizona Cancer Center, Tucson, Arizona, United States of America; 12 Department of Community Medicine and Epidemiology, Carmel Medical Center, Haifa, Israel; 13 Clalit Health Services, National Cancer Control Center, Haifa, Israel; 14 Bruce Rappaport Faculty of Medicine, Technion-Israel Institute of Technology, Haifa, Israel; Baylor University Medical Center, UNITED STATES

## Abstract

We tested for germline variants showing association to colon cancer metastasis using a genome-wide association study that compared Ashkenazi Jewish individuals with stage IV metastatic colon cancers versus those with stage I or II non-metastatic colon cancers. In a two-stage study design, we demonstrated significant association to developing metastatic disease for rs60745952, that in Ashkenazi discovery and validation cohorts, respectively, showed an odds ratio (OR) = 2.3 (P = 2.73E-06) and OR = 1.89 (P = 8.05E-04) (exceeding validation threshold of 0.0044). Significant association to metastatic colon cancer was further confirmed by a meta-analysis of rs60745952 in these datasets plus an additional Ashkenazi validation cohort (OR = 1.92; 95% CI: 1.28–2.87), and by a permutation test that demonstrated a significantly longer haplotype surrounding rs60745952 in the stage IV samples. rs60745952, located in an intergenic region on chromosome 4q31.1, and not previously associated with cancer, is, thus, a germline genetic marker for susceptibility to developing colon cancer metastases among Ashkenazi Jews.

## Introduction

Colorectal cancer is the third most common cancer and the second leading cause of cancer death in the United States, with 136,830 new cases diagnosed and 50,310 deaths annually [1]. Worldwide, colorectal cancer is the third most commonly diagnosed cancer in men, and second in women, with 746,000 cases and 614,000 cases, respectively [2].

In nearly all instances, colorectal cancer death is caused by the development of cancer metastases to distant organs, mostly commonly the liver [3]. In previous studies, we have shown that all the somatic mutations present in colon cancer liver metastases were already present in the matched antecedent colon cancer primary tumors–that is, new gene mutations are not required to initiate colon cancer metastases [4].

In this study, we examined an alternative genetic model for the development of colon cancer metastases, one which tests whether genetic variations in the human germline can confer an individual susceptibility to the metastatic spread of colon cancer. To test this model, we undertook a Genome Wide Association Study (GWAS) involving the evaluation of germline single nucleotide polymorphisms (SNPs) in Ashkenazi Jewish ancestry patients with Stage IV metastatic colon cancer compared to those with stage I or stage II colon cancers that did not metastasize. We selected the Ashkenazi Jewish population for this study because founder alleles, which are highly detectable by GWAS, are already well precedented for multiple other diseases in this population [5,6]. Using the data from our discovery cohort, we developed a preliminary power analysis that enabled us to pre-specify a limited number of SNPs whose examination in a validation cohort would provide for replication with reasonable power at the 5% significance level. The top SNPs identified in the discovery dataset were then evaluated in another population of colorectal cancer patients of Ashkenazi Jewish ancestry (validation dataset #1). Based upon the results of the discovery and validation datasets, the top SNP of interest was further evaluated in a third colorectal cancer patient population of Ashkenazi Jewish ancestry (validation dataset #2), and a combined analysis (meta-analysis) of this top SNP was completed using the results from the three Ashkenazi Jewish datasets. As a further additional test, the length of the haplotype surrounding this SNP was found to be significantly longer among the metastatic versus the non-metastatic colon cancers.

## Results

Detailed descriptions of the discovery and validation cohorts are provided in methods below. On average, the patients in the discovery study population were 71 years old, which was similar to the mean age of 72 and 65 in the validation datasets #1 and #2, respectively (Table 1). The discovery population comprised 52% males and 48% females and gender distributions in validation datasets were similar. The distributions of stage IV and stage I and II cases were also similar across the three datasets (Table 1).

**Table 1:** Characteristics of the Colon Cancer Study Populations.

	Discovery Dataset	Validation Dataset #1	Validation Dataset #2
**Sample Size**	323	462	343
**Age**			
Mean (s.d.)	71.16 (10.33)	72.24 (10.39)	64.97 (11.93)
**Gender**			
Males	169 (52.32%)	247 (53.46%)	187 (54.52%)
Females	154 (47.68%)	215 (46.54%)	156 (45.48%)
**Stage IV Colon Cancers**	89 (27.55%)	89 (19.26%)	86 (25.07%)
**Stage I & II Colon Cancers**	234 (72.45%)	373 (80.74%)^2^	257 (74.93%)
**rs60745952 (Stage I/II Cases only)**			
AA (or TT) Genotype	170 (72.65%)	254 (68.28%)	173 (67.58%)
GA (or CT) Genotype	63 (26.92%)	110 (29.57%)	77 (30.08%)
GG (or CC) Genotype	1 (0.43%)	8 (2.15%)	6 (2.34%)
MAF ^1^	0.14	0.17	0.17
**rs60745952 (Stage IV Cases only)**			
AA (or TT) Genotype	45 (50.56%)	50 (56.82%)	55 (63.95%)
GA (or CT) Genotype	35 (39.33%)	31 (35.23%)	28 (32.56%)
GG (or CC) Genotype	9 (10.11%)	8 (7.95%)	3 (3.49%)
MAF^1^	0.30	0.26	0.20
**rs60745952 (Stage I/II & IV Cases)**			
AA (or TT) Genotype	215 (66.56%)	304 (65.94%)	228 (66.67%)
GA (or CT) Genotype	98 (30.34%)	141 (30.59%)	105 (30.70%)
GG (or CC) Genotype	10 (3.10%)	16 (3.47%)	9 (2.63%)
MAF^1^ (Stage I/II & IV Cases)	0.18	0.19	0.18
MAF^1^ (Controls)	0.17	0.16	-

^1^ MAF = Minor allele frequency

^2^ rs60745952 could not be genotyped in 1 stage I/II case

**Fig 1:**
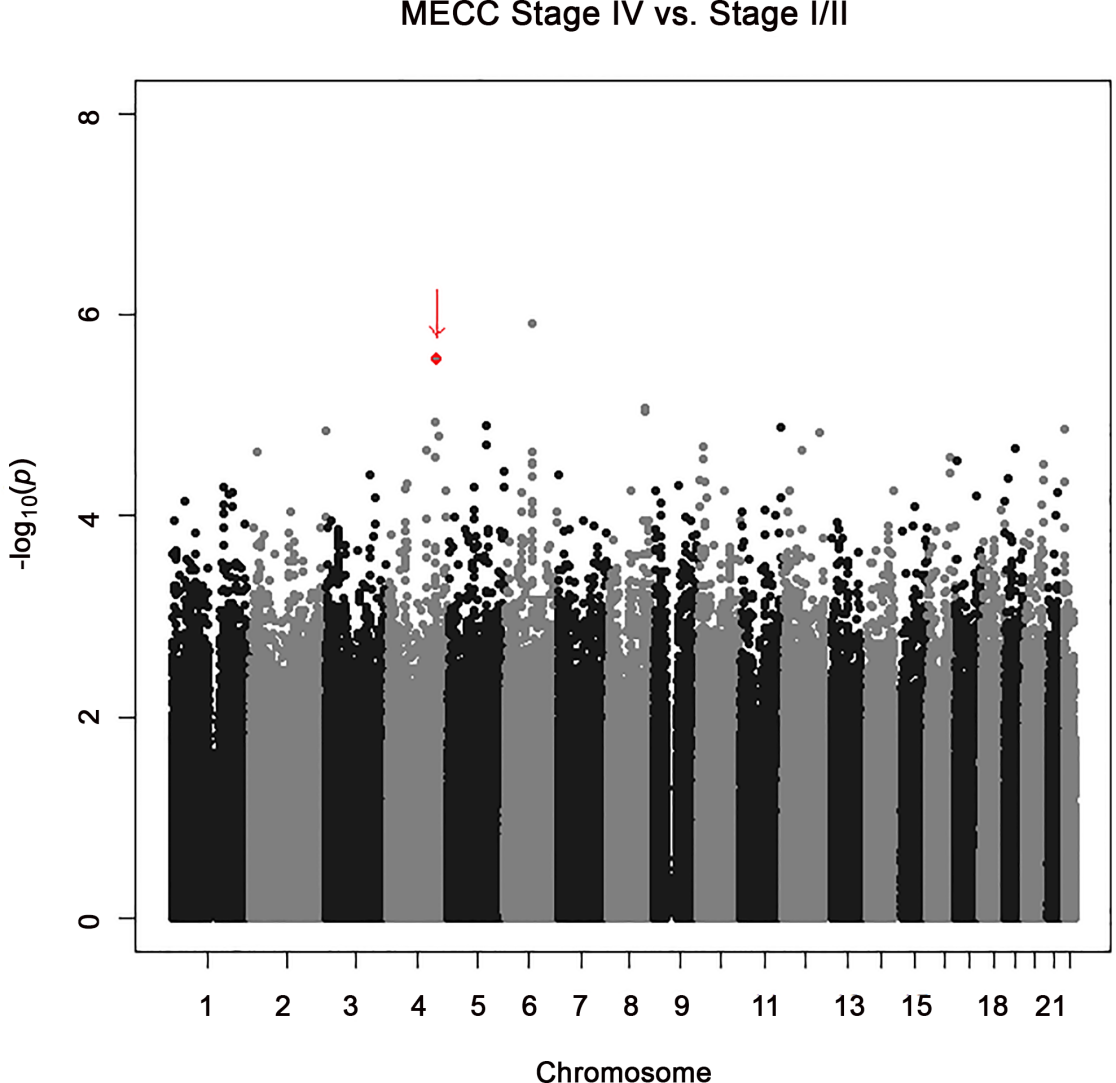
Manhattan Plot of the Discovery Dataset for Associations Between SNPs and Stage IV vs. Stage I/II Colon Cancers. The vertical axis indicates the (-log_10_ transformed) observed P-value and the horizontal axis indicates the chromosomal position of each SNP. Arrow denotes position of rs60745952 and rs72737810, which are not individually discernible at this scale of presentation.

**Fig 2:**
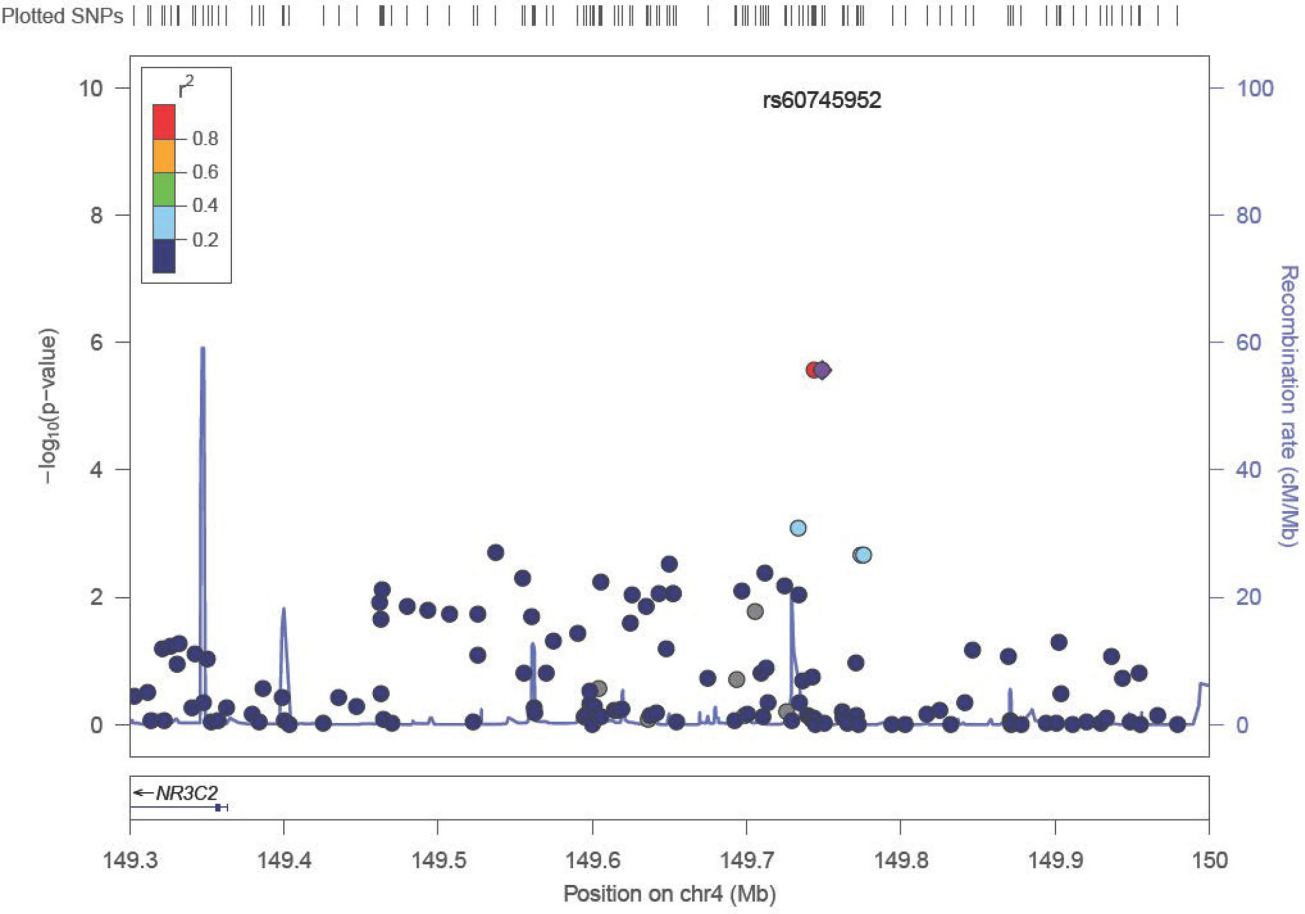
Regional Plot of loci on chromosomal region 4 (4q31.1) associated with Stage IV vs. Stage I/II colon cancer in Discovery Dataset. The horizontal axis shows SNPs along the chromosomal region and the left vertical axis shows (-log_10_ transformed) observed P-value. SNPs near the most significant SNP (rs60745952) are color coded to depict their LD with this SNP (derived as pairwise R2 values from HapMap CEU data). rs60745952, which is shown in the purple circle, is in strong LD with rs72737820, which is illustrated by the red circle. Estimated recombination rates from HapMap are plotted on the right vertical axis in cyan to reflect the local LD structure.

As shown in Table 2 and Fig 1, the most significant association observed when comparing stage IV to stage I/II colon cancer patients in the discovery GWAS was for rs2024846 on chromosome 6 (OR = 2.62; 95% CI: 1.76–3.92; p = 1.2x10^-6^). The next most significant association observed was with two SNPs (rs72737810, rs60745952) on chromosome 4q31.1 (OR = 2.83; 95% CI: 1.81–4.44; p = 2.73x10^-6^), which are separated by 5kb and are in strong linkage disequilibrium (see Fig 2). These two SNPs are located in an intergenic region distant from the nearest gene, *NR3C2*, by 380kb (Fig 2). Although the statistical significance for association for these top SNPs do not exceed standard genome-wide significance levels, a visual examination of the Q-Q plot of the observed p-values versus the expected distribution suggest that the top SNPs were worth evaluating further (S1 File), motivating us to proceed with a replication study designed to examine 20 top SNPs (Table 2) that were preselected by power for replication as described in methods below.

**Table 2:** Associations Between SNPs and Stage IV vs. Stage I/II Colon Cancers for Top 20 SNPs Selected from Discovery Dataset for Replication.

				Discovery Dataset^1^					Validation Dataset #1^1^				
SNP	Chr	BP	Nearest Gene	A1	MAF^2^	OR	95% CI	P-value	A1	MAF^2^	OR	95% CI	P-value
rs2024846	6	85575780	TBX18 | LOC100289423	A	0.20	2.62	(1.76, 3.92)	1.20E-06	A	0.27	0.90	(0.62, 1.30)	2.15E-01
rs72737810	4	149743890	NR3C2 | LOC100287246 ^3^	G	0.14	2.83	(1.81, 4.44)	2.73E-06	G	0.16	1.89	(1.27, 2.82)	9.12E-04
rs60745952	4	149748994	NR3C2 | LOC100287246	G	0.14	2.83	(1.81, 4.44)	2.73E-06	C	0.17	1.89	(1.27, 2.80)	8.05E-04
rs73351705	8	114783818	CSMD3 | TRPS1	A	0.03	5.53	(2.54, 12.05)	8.49E-06	A	0.05	1.25	(0.61, 2.57)	2.71E-01
rs77801238	8	114825951	CSMD3 | TRPS1	G	0.01	8.87	(3.27, 24.11)	9.35E-06	C	0.04	1.67	(0.79, 3.51)	8.97E-02
rs74602504	8	114823103	CSMD3 | TRPS1 ^3^	G	0.01	8.87	(3.27, 24.11)	9.35E-06	NA	NA	NA	NA	NA
rs76047883	4	149768284	NR3C2 | LOC100287246	A	0.13	2.68	(1.70, 4.23)	1.18E-05	NA	NA	NA	NA	NA
rs7737423	5	123132486	CSNK1G3 | ZNF608	A	0.04	3.78	(2.04, 7.00)	1.26E-05	NA	NA	NA	NA	NA
rs78793716	11	124941849	SLC37A2	A	0.06	3.69	(2.01, 6.77)	1.30E-05	A	0.08	0.64	(0.33, 1.27)	3.98E-01
rs13058496	22	17398812	LOC440786	G	0.23	2.18	(1.51, 3.13)	1.38E-05	G	0.24	1.20	(0.83, 1.73)	1.72E-01
rs6705378	2	240193889	HDAC4	G	0.43	2.24	(1.53, 3.26)	1.44E-05	G	0.46	0.97	(0.70, 1.34)	7.70E-02
rs11068687	12	118256497	KSR2	A	0.18	2.35	(1.58, 3.52)	1.48E-05	T	0.20	1.04	(0.70, 1.54)	4.24E-01
rs7669737	4	162361217	FSTL5	G	0.38	2.16	(1.50, 3.11)	1.59E-05	G	0.42	1.21	(0.87, 1.68)	1.26E-01
rs62377104	5	123074679	CSNK1G3 | ZNF608 ^3^	C	0.05	3.60	(1.95, 6.63)	2.00E-05	G	0.05	1.01	(0.51, 2.00)	4.87E-01
rs1556055	10	17523142	ST8SIA6 | PTPLA	G	0.20	2.40	(1.58, 3.64)	2.08E-05	G	0.24	1.03	(0.70, 1.50)	4.47E-01
rs16962543	19	29578972	LOC148145 | UQCRFS1	A	0.06	3.34	(1.87, 5.96)	2.17E-05	A	0.10	0.60	(0.31, 1.15)	4.37E-01
rs12367527	12	59398014	LRIG3 | SLC16A7	A	0.09	2.62	(1.65, 4.16)	2.26E-05	A	0.13	0.83	(0.50, 1.39)	2.55E-01
rs308420	4	123767943	FGF2	A	0.04	4.33	(2.14, 8.75)	2.27E-05	A	0.06	0.97	(0.49, 1.92)	3.76E-02
rs62451177	6	85559189	TBX18 | LOC100289423	G	0.10	2.88	(1.73, 4.80)	2.29E-05	NA	NA	NA	NA	NA
rs2203879	2	22186722	LOC100129278 | KLHL29	A	0.02	6.47	(2.63, 15.88)	2.36E-05	NA	NA	NA	NA	NA

^1^ Adjusted for age, sex and first 2 principal components (PCs); analyses conducted using a log-additive genetic model in PLINK

^2^ MAF = Minor allele (A1) frequency in Stage I/II cases; ^3^ Redundant SNPs.

**Fig 3:**
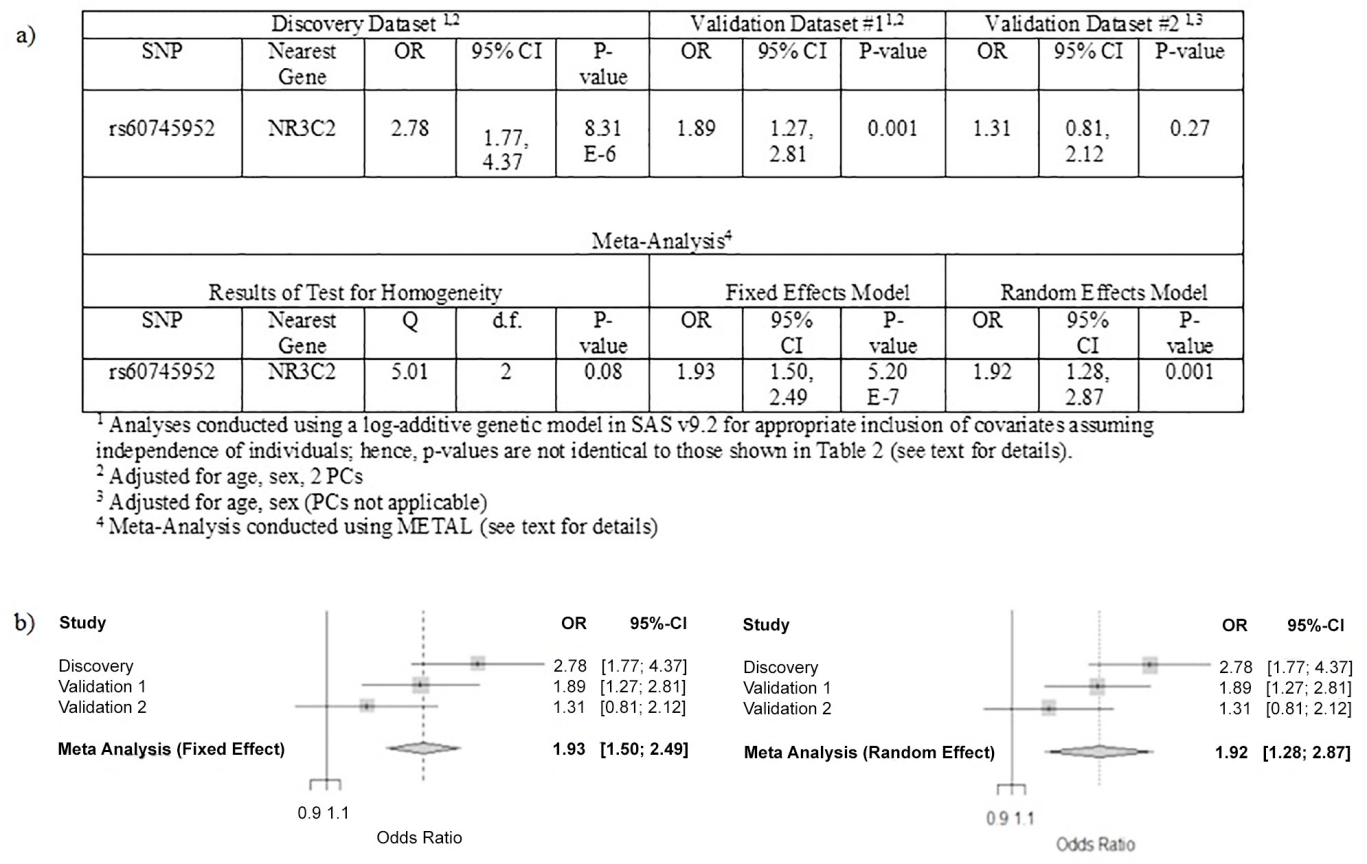
Summary of Association and Test of Homogeneity Results (a) and Forest Plots (b) for the Meta-Analysis evaluating the rs60745952 SNP and Stage IV vs. Stage I and II Colon Cancers in the Discovery and Validation #1 and Validation #2 data sets under a fixed effects and random effects model.

Table 2 lists the results observed in validation dataset #1 for the 20 SNPs preselected for testing for association with stage IV versus Stage I or II colon cancer. Replication was observed for the two SNPs at chromosome 4q31.1, rs72737810 and rs60745952. In validation data set #1, these two SNPs showed association with development of metastatic disease at p = 9.12x10^-4^ (OR = 1.89; 95% CI: 1.27–2.82) and p = 8.05x10^-4^ (OR = 1.89; 95% CI: 1.27–2.80), respectively (Table 2). The strength of association for these two correlated SNPs both exceeded P < 0.0044, which was our pre-specified significance threshold for replication (as detailed in methods). Given the strong LD between these SNPs, we arbitrarily selected rs60745952 to evaluate in the second validation dataset. In the smaller, less highly powered validation dataset #2, the association between rs60745952 and development of metastatic disease was not statistically significant, but the effect estimate was in the same direction with an increased risk for the minor allele (OR = 1.31; 95% CI: 0.81–2.12) (Fig 3, Panel A).

As the discovery dataset and both validation datasets all showed an increased risk of metastatic disease in colon or colon and rectal cancer patients who carry the minor allele of rs60745952, a meta-analysis was performed to obtain the best estimate of the overall effect size. As shown in Fig 3 (Panel B), the summary effect estimate for the combined analysis (meta-analysis) under a fixed effects model was statistically significant (OR = 1.93; 95% CI: 1.50–2.49; p = 5.20 x 10^−7^). Since we observed some evidence for heterogeneity in the test of homogeneity (Q-statistic = 5.01; d.f. = 2; p = 0.08; Fig 3, Panel A), we also evaluated the random effects model and found that the summary effect estimates were similar (OR = 1.92; 95% CI: 1.28, 2.87; p = 0.001); however, as expected, the confidence interval was tighter for the fixed effects model.

**Fig 4:**
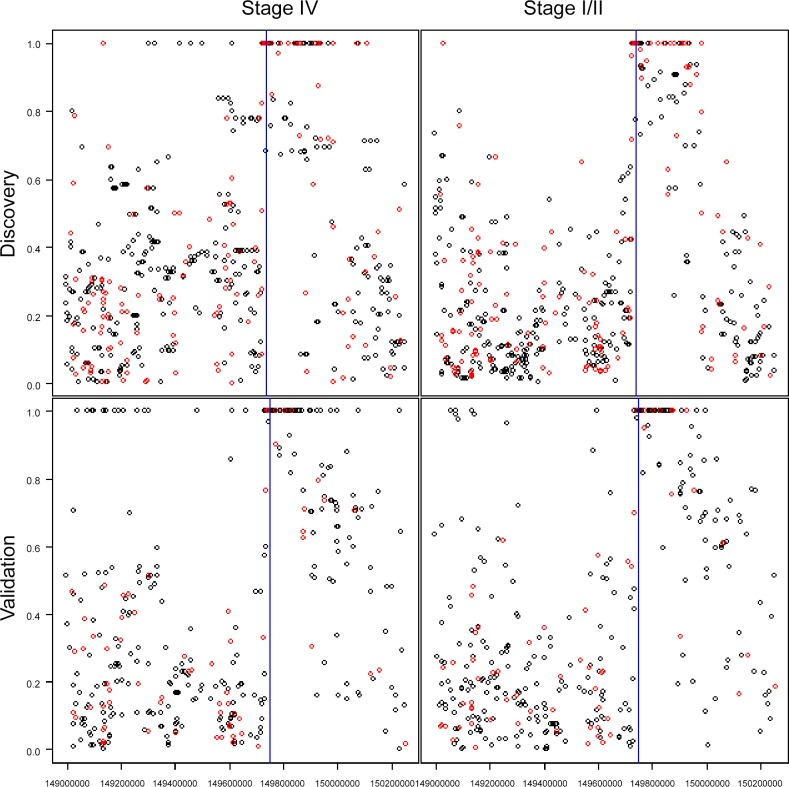
LD Plots for rs60745952 in Stage IV and Stage I/II, in Discovery and Validation #1 Datasets. Vertical axis is the LD as D’ for the rs60745952 SNP (blue line) and other SNPs up and downstream of this location available on the genotyping arrays. Red dots indicate SNPs that were on the genotyping arrays in both the Discovery Dataset and Validation Dataset #1.

We further reasoned that, if association of rs6074592 to risk of developing metastatic disease was driven by linkage disequilibrium with a founder metastasis susceptibility allele, and if this founder allele had arisen more recently than the Ashkenazi population as a whole, this allele would be segregating on a haplotype block of increased size present in the metastasis versus non-metastasis associated genomes. To test for this possibility, we examined the LD structure of the region near the rs60745952 SNP in individuals with stage IV versus stage I or II colon cancers. Fig 4 shows plots of the D’ between rs60745952 (blue line) and all other SNPs in the region. The clustering of SNPs with D’ = 1 to rs60745952 defines a haplotype that is clearly longer in individuals with stage IV compared to stage I/II colon cancers in both the discovery and the validation #1 datasets (red circles in Fig 4 indicate the SNPs in common in both discovery and validation # 1 datasets). More specifically, we observed that the number of SNPs with D’ = 1 in stage IV (58, permutation variance = 11.71) was significantly greater than that observed in stage I/II (41, permutation variance = 7.60) in the discovery dataset (p = 2.73×10–5) (Table 3; S4 File). Results in the validation #1 dataset were similar, only a little less significant (p = 5.00×10–5).

**Table 3:** Number of SNPs with D’ = 1.0 in a Selected Chromosomal Region for Stage IV and Stage I/II Colon Cancers in Discovery and Validation #1 Datasets.

	Stage I/II		Stage IV		Difference		
	Observed	S.D.	Observed	S.D.	Observed	S.D.^1^	P-value^2^
Discovery	41	2.76	58	3.42	17	4.39	2.7 x 10^−5^
Validation Dataset #1	52	1.23	65	3.27	13	3.50	5.0 x 10^−5^

^1^ SD = √(V_1_+V_2_), V_1_ and V_2_ were estimated from the permutation distribution. See S4 File for details.

^2^ P-value from a one-sided test.

In addition, we examined the potential association between rs6074592 and Stage IV versus Stage I/II colon cancer in a Caucasian population of colon cancer patients from Kentucky and found that the association was not statistically significant (OR = 0.89; 95% C.I.: 0.55–1.46; p = 0.65), which suggests the association between rs6074592 and metastatic colon cancer is specific to patients with Ashkenazi Jewish ancestry.

## Discussion

To our knowledge, this is the first study to report genetic associations of human germline variants with the risk of developing colon cancer metastasis, specifically in this study observing that, among Ashkenazi Jewish individuals with colon cancer, carriage of the minor allele for rs60745952 was significantly associated with developing stage IV disease, with distant organ metastases, versus localized stage I or II disease, with an OR of nearly 2. The minor allele for rs60745952 is carried by 31% of the general Ashkenazi population, and by up to approximately 50% of Ashkenazi individuals with stage IV colon cancer, and hence is a major contributor to the development of colon cancer metastasis in this population. The likelihood that there is in this population a founder metastasis susceptibility variant in strong linkage disequilibrium (LD) with rs60745952 is independently suggested by observing that in Ashkenazi individuals with stage IV colon cancer, rs60745952 lies in an LD block that is significantly longer than is observed in individuals with non-metastatic stage I or II disease. We further believe the association of rs60745952 with metastatic disease is likely specific to the Ashkenazi Jewish population, as this association was not observed when evaluated in the general Caucasian population of colon cancer patients from Kentucky. In light of the very different evolutionary history of the Ashkenazi Jewish population, the differences observed are not surprising.

Previous studies from our group [4] demonstrated that all of the somatic mutations detected in colon cancer metastases were already present in their antecedent primary colon cancer tumors. Thus colon cancer metastases did not require acquisition of new “metastasis driver” mutations. In this study, we present evidence that one genetic element of metastasis progression may be encoded in the host genome. While speculative, possible host encoded factors relevant to the metastatic process would include elements of immune surveillance, elements of vascular permeability and/or angiogenesis, and conceivably elements within signaling cascades that could determine cellular responses to activated oncogenes or inactivation of tumor suppressor genes. Similar to the findings of many GWAS studies, rs60745952 lies in an intergenic region, and we do not have direct evidence of functional differences associated with carriage of this SNP. Future investigations of this question will need to be carried out in biological samples obtained specifically from the Ashkenazi population.

The findings reported here would likely not have come to light except for our employing a power guided design for replication that enabled us to preserve sufficient power to demonstrate significance of association even in a modest size replication cohort. This approach will likely be of value to other investigators who attempt to genetically enrich for an association signal by studying selected, but smaller populations, than those that have typically been employed for GWASs. Testing for differences in LD structure consistent with founder disease variants may also be of value for GWASs in these smaller, but highly selected populations.

## Materials and Methods

### Study Participants

#### Overview

Colon cancer patients from four different datasets were used in this evaluation. All patients were ascertained under research protocols approved by the Institutional Review Boards of the corresponding institutions (Carmel Medical Center (Haifa), University of Southern California, Memorial Sloan-Kettering Cancer Center and University Hospitals Case Medical Center), as detailed further below. All patients provided informed written consent. All consent procedures were approved by the corresponding aforementioned Institutional Review Boards.

#### Discovery Dataset

Participants were drawn from the Molecular Epidemiology of Colorectal Cancer study (MECC), which is a population-based case-control study of incident colon and rectal cancer cases that has been described previously [7]. Briefly, patients from northern Israel have been recruited into MECC since 1998. As part of the MECC study, basic demographic and clinical information as well as blood samples were obtained. Cases selected for genotyping had histologically confirmed, microsatellite stable colon cancer. Specifically, the study population used in the discovery dataset for this evaluation consisted of 89 individuals with stage IV colon cancer with distant organ metastases, all involving the liver, and 234 individuals with stage I or stage II colon cancers that were organ confined and without metastatic spread. These individuals were further confirmed as remaining metastasis-free during clinical follow-up of at least 3 years’ duration. Controls in the MECC discovery dataset study population consisted of individuals with no prior history of colon or rectal cancer and were individually-matched to cases based on age, gender, and primary clinic site. 139 controls were genotyped and used to quantify the minor allele frequency for top SNPs. All patients had self-reported Ashkenazi Jewish ancestry.

#### Validation Dataset #1

The first validation dataset was derived from a second independent sample of the MECC study participants that were selected with less restrictive criteria than in the discovery dataset. Specifically, validation dataset #1 comprised further individuals with either colon or rectal cancer, stage I and II cases for whom post-operative follow-up was not available, and individuals whose cancers had not been tested for microsatellite instability. In addition, this sample included individuals with self-reported Sephardic as well as Ashkenazi Jewish ancestry. The validation dataset in this evaluation included 89 stage IV colorectal cancer cases and 373 stage I and stage II colorectal cancer cases. Controls in the validation dataset #1 study population consisted of further patients with no prior history of colon or rectal cancer and were individually-matched to cases based on age, gender, Jewish ethnicity and primary clinic site.

#### Validation Dataset #2

Patients in the second validation dataset were treated at Memorial Sloan-Kettering Cancer Center (MSKCC) and had histologically confirmed colorectal cancer. Patients were ascertained under two MSKCC clinical protocols between 2000 and 2010. This study population was also less restrictive than the discovery dataset in that validation dataset #2 included individuals with either colon or rectal cancer and individuals whose cancers had not been tested for microsatellite instability. This validation dataset consisted of 86 individuals with stage IV colon cancer with distant organ metastases, all involving the liver, and 256 individuals with stage I or stage II colon or rectal cancers that were organ confined and without metastatic spread. Individuals with these early stage cancers were further confirmed as remaining metastasis free during clinical follow-up of at least 3 years’ duration. All patients had self-reported Ashkenazi Jewish ancestry.

#### Kentucky Caucasian Dataset

The details of the Kentucky study population have been described elsewhere [8]. Briefly, the dataset used in this evaluation consisted of 77 patients with Stage IV colon cancer and 398 patients with Stage I/II colon cancer. All patients in this sample were Caucasian.

### DNA Isolation, Genotyping, and Quality Control

#### Discovery Dataset

DNA from whole blood lymphocytes was extracted using the QIAamp DNA Blood Mini Kit (Qiagen, Hilden, Germany) and was stored at −20°C until use for genotyping. All DNA samples underwent whole genome amplification using the Illustra Genomiphi V2 DNA Amplification Kit (GE Healthcare, Waukesha, Wisconsin; catalog No. 25660032). The samples were stored at −20°C.

Samples were genotyped on the Illumina Omni 2.5–8 platform. Data were cleaned using quality control procedures recommended by the eMERGE Genomics Working Group [9]. The QC procedures include evaluation of sample and marker call rate, gender mismatches (PLINK [10]; “—check-sex”), duplicates, Hardy-Weinberg equilibrium, sample relatedness (PLINK [10]; “—genome”), and population stratification. A total of 1,491,783 SNPs were included in the analysis after excluding SNPs that had minor allele frequencies <0.01, missing data in greater than 2% of samples, or Illumina GenTrain scores less than 0.6. In total, 5 stage IV patients, and 7 stage I and II patients, were removed from the analyses for having call rates less than 98%, for failing the gender check or for being an unintended duplicate (see S1 File for additional details). To control for population stratification, the first two principal components (PCs) were derived from multidimensional decomposition analysis using the ‘cmdscale’ function in R.

#### Validation Dataset #1

The Colorectal Transdisciplinary (CORECT) Study conducted the genotyping for validation dataset #1. Genotyping was conducted using a custom Affymetrix genome-wide platform (the Axiom® CORECT set) with approximately 1.3 million SNPs and indels; however, only data from SNPs identified as the Top SNPs in the discovery dataset were extracted by CORECT for this analysis (see Statistical Analyses). Data were cleaned using quality control procedures similar to the discovery dataset (see S2 File for details) [9].

#### Validation Dataset #2

DNA was isolated from either whole blood lymphocytes or from formalin fixed paraffin embedded (FFPE) normal colonic tissue using Qiagen QIAamp DNA extraction kit or the QIAamp DNA Blood Mini Kit (Qiagen, Hilden, Germany) and was stored at −20°C until use for genotyping. All DNA samples underwent whole genome amplification using the Repli-g DNA Amplification Kit (Qiagen, CA). For a subset of samples, both FFPE and blood derived DNA were available and were used to test concordance of genotypes for quality assurance. Genotyping for rs60745952 was performed using Sequenom iPLEX assays and analyzed using MassARRAY [11]. Genotypes were called using TYPER 4.0.2 software.

#### Kentucky Caucasian Dataset

DNA was isolated from all subjects and ABI TaqMan chemistry was employed to genotype rs60745952. This required custom design of probes and primers by ABI. PCR [40 Cycles] was carried out on an ABI GeneAmp PCR System 9700 Dual Head Instrument and endpoint reads were performed using the ABI 7900 Sequence Detection System.

### Statistical Analyses

Logistic regression was conducted to evaluate the potential association between each SNP and stage IV versus stages I and II colon cancers under a log-additive genetic model. Although initial analyses used PLINK [10], the final analysis for rs60745952 in the three study datasets used SAS v9.2 (SAS Institute Inc., Cary, NC) for appropriate inclusion of covariates assuming only independence of individuals, not of alleles [12] as implemented in PLINK [10]. All regression analyses were adjusted for age and gender and, in the case of the discovery and validation dataset #1, which were GWAS, the first 2 principal components. Furthermore, in these two datasets we examined whether rs60745952 was carried on a longer haplotype in stage IV patients than in stage I/II patients. After identifying the appropriate chromosomal region in the discovery data set using the LD pattern generated in Haploview [13] (S3 File), we calculated the D’ between each SNP in the block and rs60745952 and counted the number of SNPs for which D’ = 1. We then performed permutation testing to test whether the number of SNPs with D’ = 1 was significantly larger in the stage IV compared to the stage I/II patients (S4 File).

### Design for Replication Studies

Statistical modeling suggested that a replication cohort of colon cancer cases of similar size to the discovery cohort would provide about 80% power to test up to 9 of our most significant candidate SNPs (top SNPs) at a Bonferroni corrected significance level of 0.04/9 (= 0.0044), plus 8 further SNPs at a Bonferroni corrected significance level of 0.01/8; thus the overall type 1 error was controlled to be 0.05 (A similar design was used to test stage III versus stage I/II colon cancer patients, but only the comparison with stage IV patients is reported here). Power for replication was calculated for each individual SNP based on the 50% lower confidence limit of the corresponding odds ratio [14] and the prevalence of the SNP (see S5 File). Three of the top 9 SNPs were accompanied by a companion SNP that demonstrated complete linkage disequilibrium in our discovery cohort (D’ = 1.0), and these 3 companion SNPs were added to our set of top SNPs to be tested for replication, with no statistical adjustment, for a total of 20 SNPs pre-specified for analysis in the validation cohort. Prior to initiating the validation study, this analysis design and the identities of the 20 pre-specified SNPs were deposited with the NCI program officer overseeing the study to record documentation of the study design. Additional SNPs that were present on the validation dataset #1 genotyping array were not considered in this study evaluation.

### Combined Data Analysis (Meta-Analysis)

The association results for the rs60745952 SNP in the three study datasets with Ashkenazi Jewish ancestry (Discovery Dataset, Validation Dataset #1, Validation Dataset #2) were combined using a meta-analysis with the inverse sample size-weighted approach implemented in METAL [15]. We evaluated homogeneity between the association results using the Q-test and calculated the summary effect estimate using a random effects model [15]. We calculated the summary effect estimate using both fixed and random effect models, since the p-value for the test of homogeneity was marginally significant (Table 3; p<0.10), which suggests some heterogeneity may be present and the variance between studies should be incorporated into the analyses to obtain confidence limits.

## Supporting Information

S1 File Genotyping QA/QC Details for Discovery Dataset. (PDF)

S2 File Genotyping QA/QC Details for Validation Dataset #1. (PDF)

S3 File LD Plots. (PDF)

S4 File LD Evaluation through Permutation in Discovery and Validation #1 Datasets. (PDF)

S5 File Selecting SNPs for Validation. (PDF)
