# Supplementary material for: A Germline Variant on Chromosome 4q31.1 Associates with Susceptibility to Developing Colon Cancer Metastasis
Source: PLoS One. 2016 Jan 11;11(1):e0146435. doi: 10.1371/journal.pone.0146435 (PMC4709047; doi:10.1371/journal.pone.0146435)

S1 FILE:

GENOTYPING QA/QC DETAILS FOR DISCOVERY DATASET

We performed the following QA/QC procedures:

1. Gender check:

Gender check was performed in PLINK using the "--check-sex" option. 9 females were misclassified as males, 3 males were misclassified as females and 6 individuals had no obvious sex based on their heterozygosity on the X chromosome.

2. Sample relatedness check:

Sample relatedness check was performed in PLINK using the "--genome" option. There were 42 pairs of individuals with kinship coefficients greater than 0.05. We plotted the figure for Z1 (the probability of a pair of individuals with IBS=1) vs. Z0 (the probability of a pair of individuals with IBS=0). Before we excluded 38 individuals, there were some outliers and suspect relatives in the plot. After we excluded 38 individuals, the plot showed that the relatives had been removed.

Table S1. No. of Individuals in Stages

	Stage I/II	Stage IV
Before removing 38 Individuals	247	94
After removing 38 Individuals	234	89

3. There were 2,379,855 SNPs in total. We removed SNPs in which the minor allele frequency was less than 1%. Then 816,368 SNPs were excluded and 1,563,220 SNPs remained.

4. SNPs with missing rates >2% were excluded. Then 20,279 additional SNPs were excluded.
5. SNPs with GenTrain Score in Illumina<0.6 were excluded. Then 51,581 additional SNPs were excluded in this step and 1,491,783 SNPs remained.

S1 Figure 1. Q-Q Plot of Discovery Dataset for Stage IV vs. Stage I/II Colon Cancers. The vertical axis indicates (-$\log_{10}$ transformed) observed P-value and the horizontal axis indicates (-$\log_{10}$ transformed) expected P-value. The rs60745952 SNP is shown in red with red arrow.

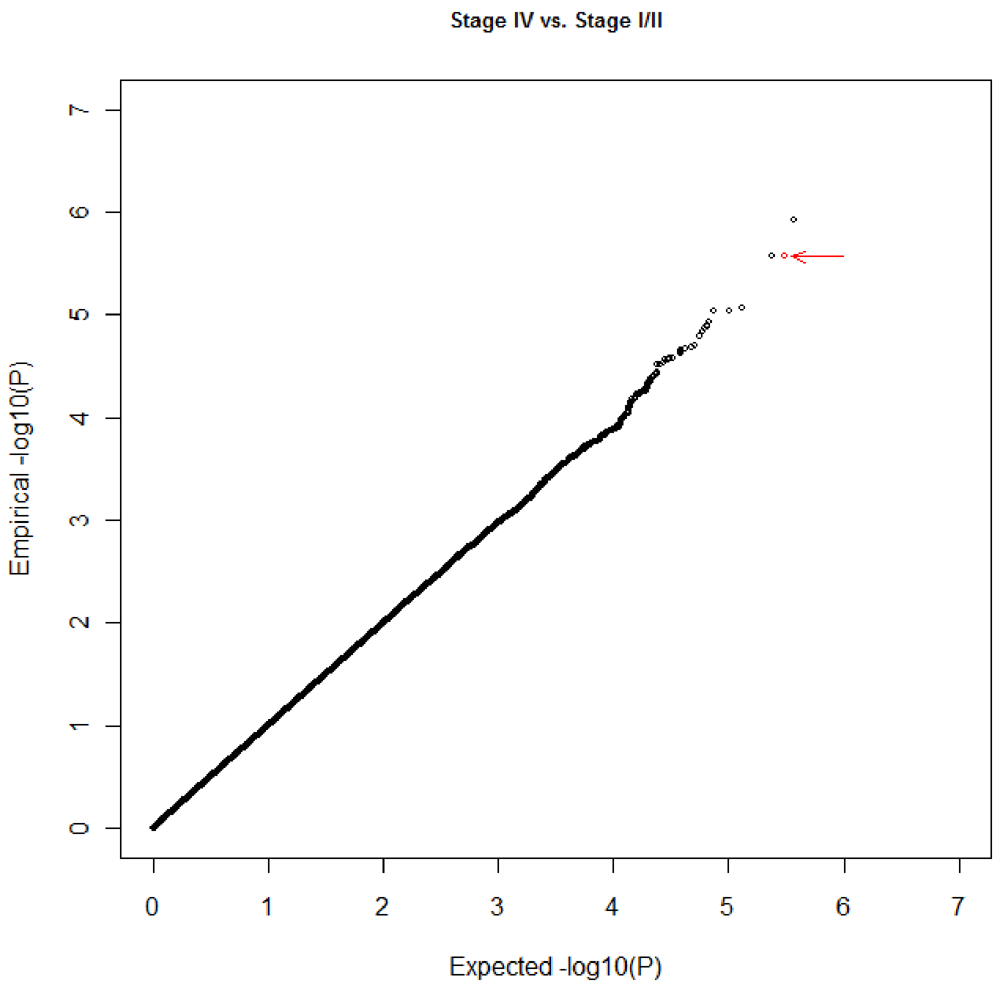

Supplement: S1 File — (PDF) [file pone.0146435.s001.pdf]
