# Supplementary material for: A Germline Variant on Chromosome 4q31.1 Associates with Susceptibility to Developing Colon Cancer Metastasis
Source: PLoS One. 2016 Jan 11;11(1):e0146435. doi: 10.1371/journal.pone.0146435 (PMC4709047; doi:10.1371/journal.pone.0146435)
Supplement: S2 File — (PDF) [file pone.0146435.s002.pdf]

S2 FILE:

GENOTYPING QA/QC DETAILS FOR VALIDATION DATASET #1

The Colorectal Transdisciplinary (CORECT) study provided the infrastructure and support for genotyping and cleaning of the data. Genotyping was conducted using a custom Affymetrix genome-wide platform (the Axiom® CORECT Set) with approximately 1.3 million SNPs and indels on two physical genotyping chips. Genotype data were cleaned based on quality control (QC) metrics at the individual subject and SNP levels. Samples with <95% call rate, sex mismatches (between self-reported and genotypic predicted sex), low concordance with previous genotype data, duplicate samples, unanticipated genotype concordance, identity-by-descent (IBD) with another sample, or ethnic outliers as identified by visual inspection of principal components analysis cluster plots were removed. SNPs with <95% call rate, concordance <95% with 1000 Genomes in samples genotyped for quality control, or HWE $p<10^{-4}$ in controls were excluded. Called markers were used to evaluate LD (in Figure 4 and Supplemental Materials, S4).

References:

1000 Genomes Project Consortium (Altshuler, D) et al. (2010). A map of human genome variation from population-scale sequencing. Nature. 467:1061-73.
