# Supplementary material for: A Germline Variant on Chromosome 4q31.1 Associates with Susceptibility to Developing Colon Cancer Metastasis
Source: PLoS One. 2016 Jan 11;11(1):e0146435. doi: 10.1371/journal.pone.0146435 (PMC4709047; doi:10.1371/journal.pone.0146435)

S3 File:

LD PLOTS FROM HAPLOVIEW FOR STAGE IV AND STAGE I/II COLON CANCER PATIENTS IN THE DISCOVERY DATASET

DISCOVERY DATASET: STAGE IV LD PLOT FROM HAPLOVIEW

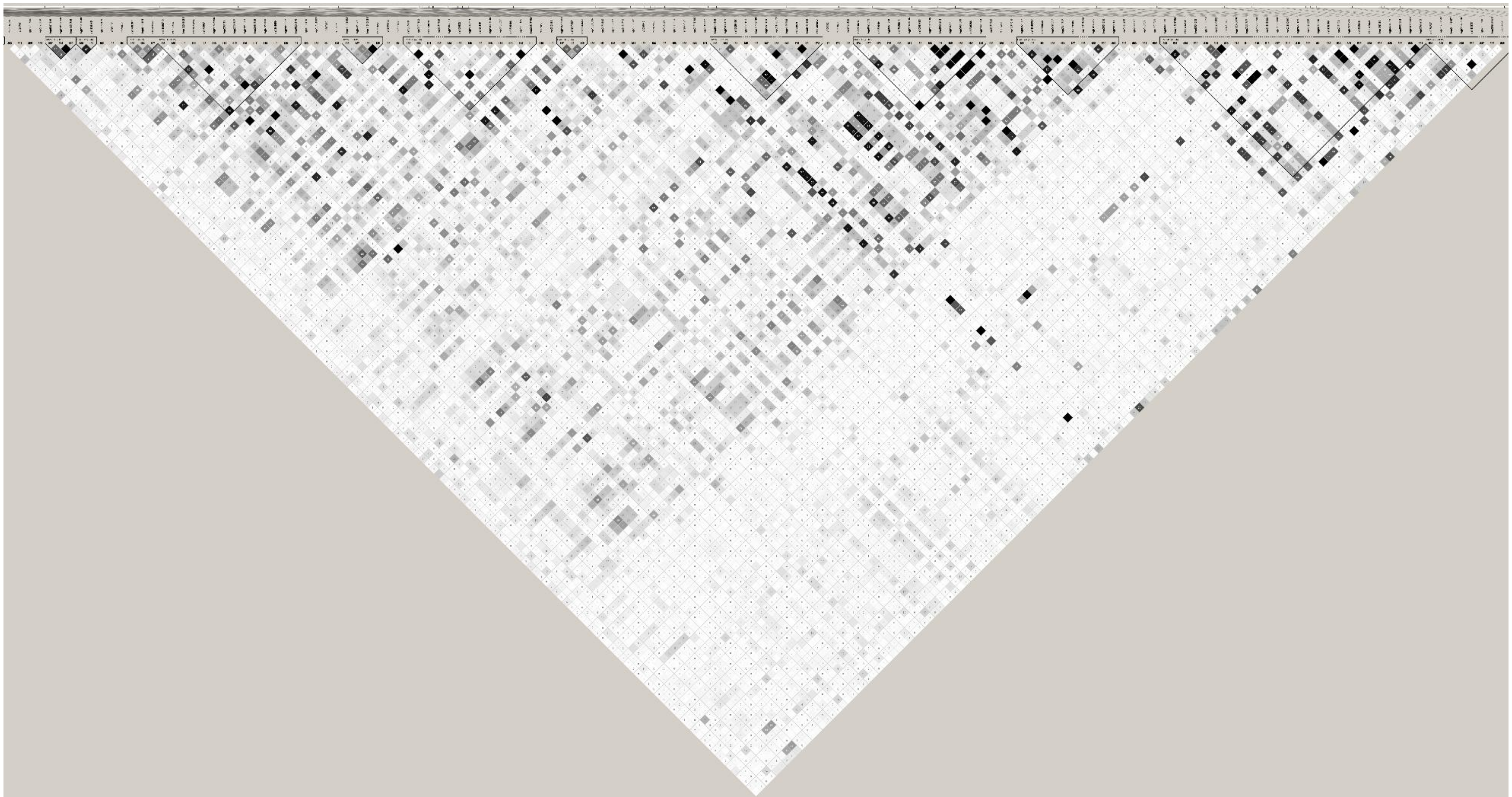

DISCOVERY DATASET: STAGE I/II LD PLOT FROM HAPLOVIEW

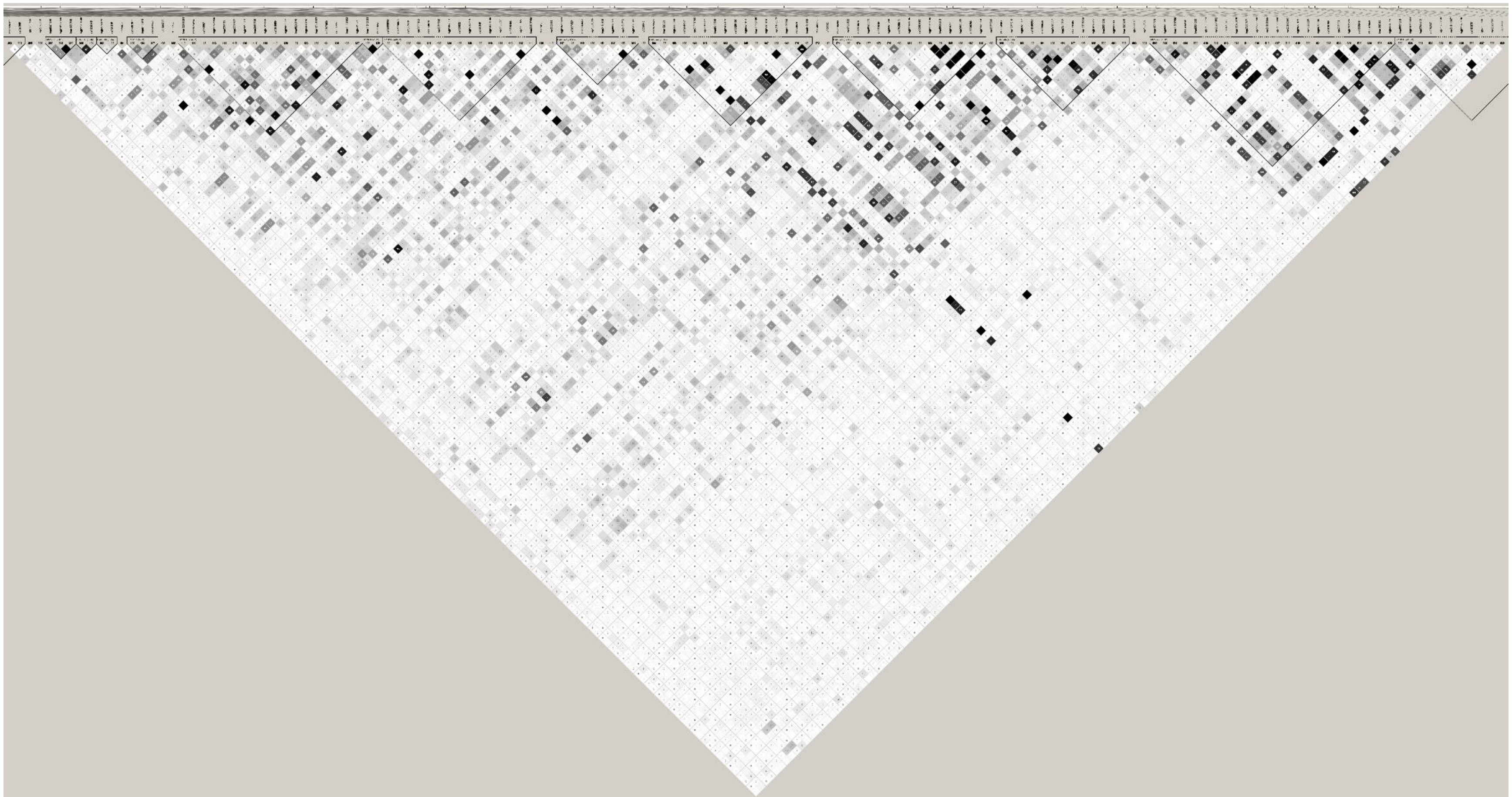

Supplement: S3 File — (PDF) [file pone.0146435.s003.pdf]
