# Supplementary material for: A Germline Variant on Chromosome 4q31.1 Associates with Susceptibility to Developing Colon Cancer Metastasis
Source: PLoS One. 2016 Jan 11;11(1):e0146435. doi: 10.1371/journal.pone.0146435 (PMC4709047; doi:10.1371/journal.pone.0146435)
Supplement: S4 File — (PDF) [file pone.0146435.s004.pdf]

S4 File:

LD EVALUATION THROUGH PERMUTATION IN DISCOVERY AND VALIDATION #1 DATASETS

We want to estimate the null distribution of the number of SNPs for which D’=1 in the region without assuming independence of the SNPs. We generated 200 replicate permutations for each group (stages I/II and IV) to obtain the null distributions. For each individual and each SNP, separately, we permuted the genotype. The permutations were performed as follows:

The minor allele frequencies were kept exactly the same as in the original data.
Missing genotypes were allowed and randomized as genotypes in the permutations.
D’ between rs60745952 and each of the other SNPs was calculated.

We then counted, for each permutation, the number of SNPs in the region for which D’=1 to obtain the empirical null distribution for the number of SNPs in the region for which D’=1.
The empirical null distributions for Stage I/II and Stage IV in the discovery and validation datasets are shown below as histograms:

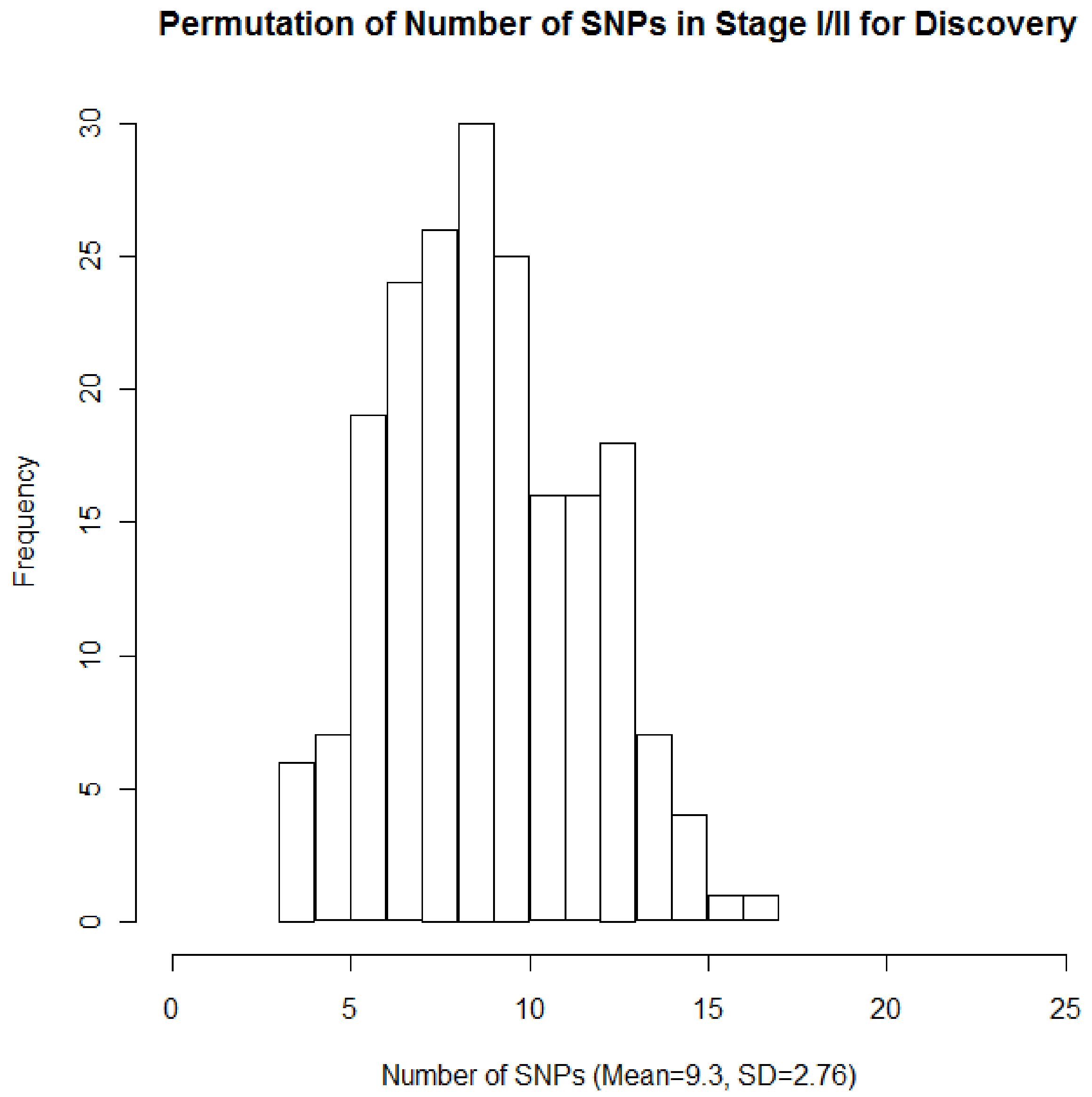

Frequency distribution of the number of SNPs with D'=1 relative to rs60745952 as determined in 200 permutations of stage I/II cases in the discovery dataset.

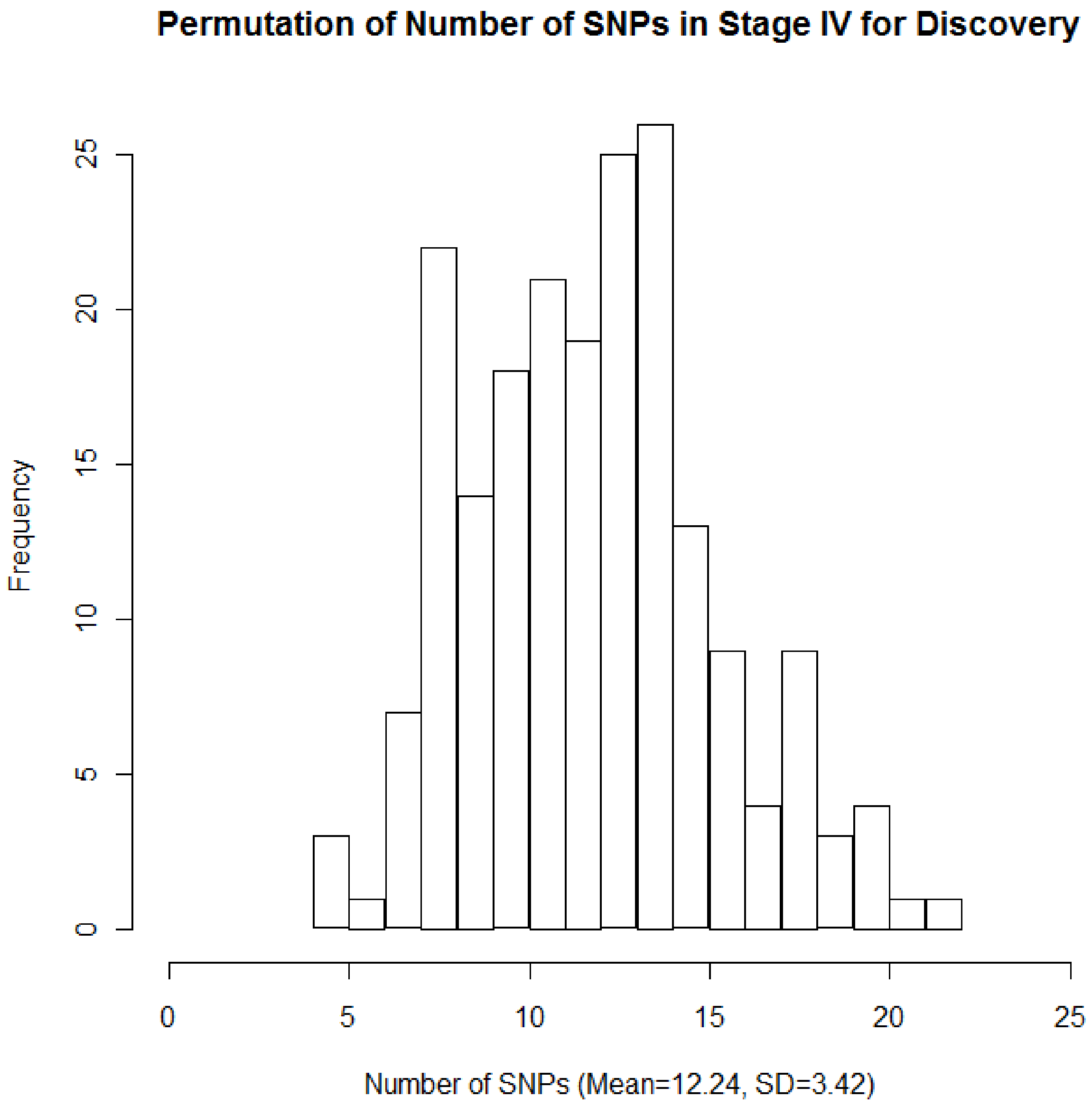

Frequency distribution of the number of SNPs with D’=1 relative to rs60745952 as determined in 200 permutations of stage IV cases in the discovery dataset.

The empirical null distributions for Stage I/II and Stage IV in the Validation dataset #1 are shown below:

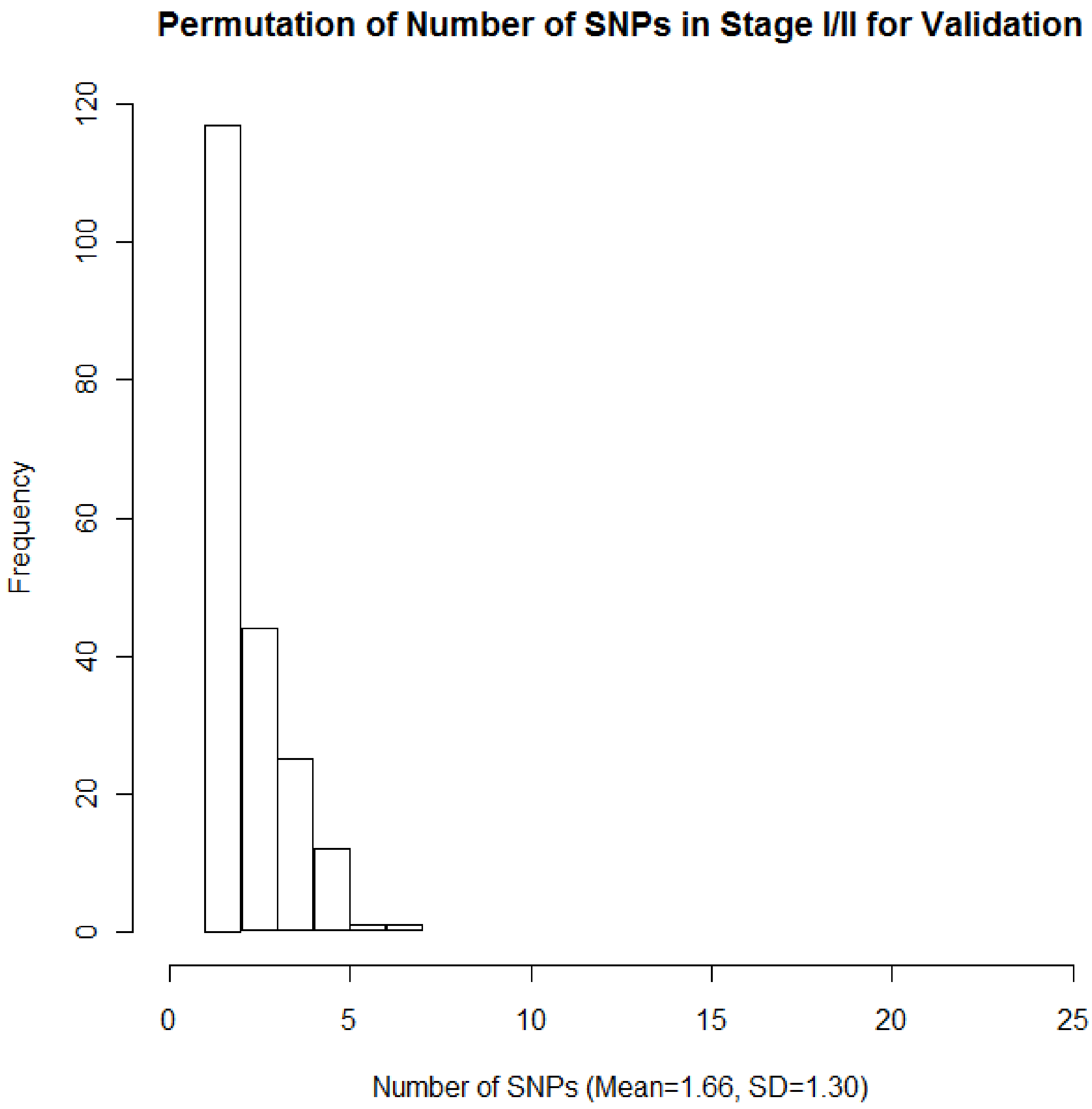

Frequency distribution of the number of SNPs with D’=1 relative to rs60745952 as determined in 200 permutations of stage I/II cases in the validation dataset #1.

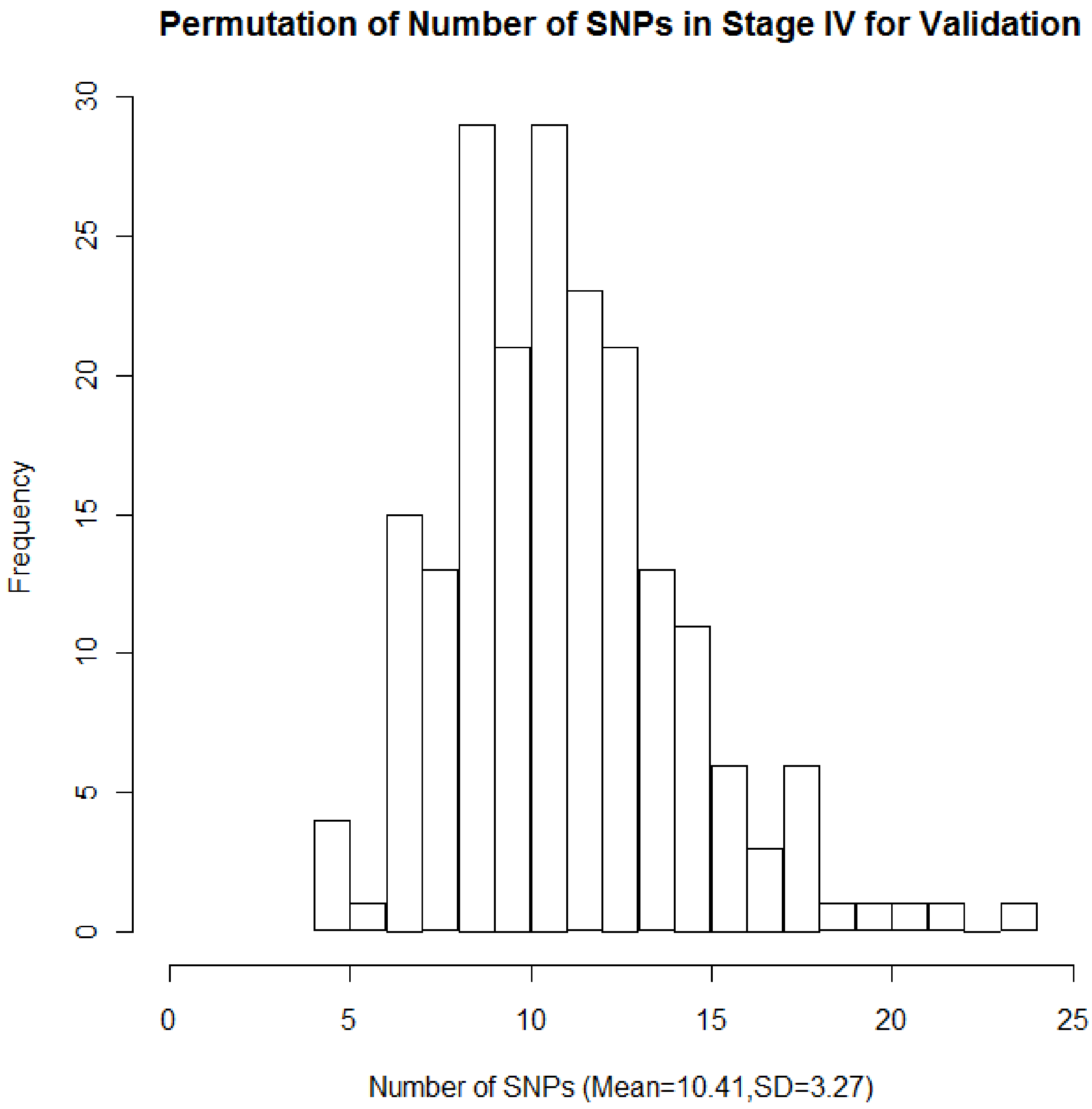

Frequency distribution of the number of SNPs with D'=1 relative to rs60745952 as determined in 200 permutations of stage IV cases in the validation dataset #1.

We performed a test between the two groups of the number of SNPs for which D'=1 based on the following assumptions:

The null distribution of the permutation statistic is normally distributed.

We have a sufficiently large number of permutation so that we can use a z score to perform the test (i.e. assume normality, with the permutation variance estimate, of the number of SNPs in the region for which D'=1).

The observed count is denoted y. The variance from the permutation distribution is denoted V. The two groups are independent. Thus the variance of the difference between the two observed numbers is the sum of the variances, i.e. the variance of the difference is (V_1+V_2), letting 1 denote stages I/II and 2 denote stage IV. We perform a one-sided test of the observed group difference. The corresponding z-statistic is $(y_2-y_1)/\sqrt{(V_1+V_2)}$. Table 3 in the text shows y, $SD=\sqrt{V}$, y_2-y_1 and $SD=\sqrt{(V_1+V_2)}$. Except for the stage I/II validation dataset, the distributions are approximately normally distributed. The skewness of that distribution would tend to make the P-value conservative, i.e. too large. To verify that the assumption of normality was not critical, for each dataset we estimated the null distribution of the z-statistic as follows. We simulated 32 replicates from each group, noting the observed count and variance. We then paired each replicate from the stage I/II data with each replicate from the stage IV data to calculate 1024 replicate z-statistics. For each dataset, the z-statistic calculated from the observed counts given in Table 3 was larger than any of the simulated null distribution z-statistics, thus demonstrating that the p-values are robustly $< 10^{-3}$.
