# Supplementary material for: A Germline Variant on Chromosome 4q31.1 Associates with Susceptibility to Developing Colon Cancer Metastasis
Source: PLoS One. 2016 Jan 11;11(1):e0146435. doi: 10.1371/journal.pone.0146435 (PMC4709047; doi:10.1371/journal.pone.0146435)
Supplement: S5 File — (PDF) [file pone.0146435.s005.pdf]

S5 File:

SELECTING SNPS FOR VALIDATION

We selected 20 SNPs from among the top SNPs to have good power in the validation sample; we ended up with 17 independent SNPs.

1. We calculated the 50% lower confidence interval (LCI) for odds ratio of the top SNPs in the discovery dataset.

2. We modeled a validation population that, unlike the discovery cohort, would not exclude individuals with tumors that had microsatellite instability (MSI). We hence modeled the validation population by assuming that MSI cases would constitute

22% of Stage I/II cases and 10% of Stage IV cases.

Since the MSI pathway is biologically distinct, we further assumed that it would not interact with our disease allele, and that the allele frequencies in MSI cases would correspond to population controls. Using these modeled allele frequencies, we derived the crude odds ratios OR_{new}.

3. Then, to calculate the power, we used as effect size the 50% lower confidence limit (LCI) of the OR originally calculated from the discovery dataset with the inclusion of covariates, but adjusted to allow for the different allele frequencies: $\text{ajusted LCI} = \text{estimated LCI} \times \frac{OR_{new}}{OR_{old}}$, where OR_{old} is the crude odds ratio based on the allele frequencies in the discovery dataset.

4. We assumed LD=1 and disease prevalence=$2p-p^2$, where p is the modeled allele frequency of Stage I/II.

5. Then, knowing the number of stage I/II and stage IV cases in the validation dataset, we used Purcell's on-line Genetic Power Calculator. (http://pngu.mgh.harvard.edu/~purcell/gpc/) to calculate the power (Purcell S, 2003).

6. We selected the top 20 SNPs that had at least 80% power.

Reference:

Purcell S, C. S. (2003). Genetic Power Calculator: Design of linkage and association genetic mapping studies of complex traits. *Bioinformatics*, 19: 149-150.
